# Exploration of the Potential Relationship Between Gut Microbiota Remodeling Under the Influence of High-Protein Diet and Crohn’s Disease

**DOI:** 10.3389/fmicb.2022.831176

**Published:** 2022-03-03

**Authors:** Yiming Zhao, Lulu Chen, Liyu Chen, Jing Huang, Shuijiao Chen, Zheng Yu

**Affiliations:** ^1^Department of Gastroenterology, Xiangya Hospital, Central South University, Changsha, China; ^2^Department of Microbiology, School of Basic Medical Science, Central South University, Changsha, China; ^3^National Clinical Research Center for Geriatric Disorders, Xiangya Hospital, Central South University, Changsha, China; ^4^Department of Parasitology, School of Basic Medical Science, Central South University, Changsha, China

**Keywords:** Crohn’s disease, tryptophan, *Helicobacter*, microbial interaction, high-protein diet, gut microbiota

## Abstract

Diet and gut microbiota are both important factors in the pathogenesis of Crohn’s disease, and changes in diet can lead to alteration in gut microbiome. However, there is still insufficient exploration on interaction within the gut microbiota under high-protein diet (HPD) intervention. We analyzed the gut microbial network and marker taxa from patients with Crohn’s disease in public database (GMrepo, https://gmrepo.humangut.info) combined with investigation of the changes of composition and function of intestinal microbiome in mice fed on HPD by metagenomic sequencing. The results showed that there was an indirect negative correlation between *Escherichia coli* and *Lachnospiraceae* in patients with Crohn’s disease, and *Escherichia coli* was a marker for both Crohn’s disease and HPD intervention. Besides, enriched HH_1414 (one of the orthologs in eggNOG) related to tryptophan metabolism was from *Helicobacter*, whereas reduced orthologs (OGs) mainly contributed by *Lachnospiraceae* after HPD intervention. Our research indicates that some compositional changes in gut microbiota after HPD intervention are consistent with those in patients with Crohn’s disease, providing insights into potential impact of altered gut microbes under HPD on Crohn’s disease.

## Introduction

Crohn’s disease is a subtype of inflammatory bowel diseases (IBDs) characterized by chronic inflammation of any part of the gastrointestinal tract, with a progressive and destructive course as well as an increasing incidence worldwide (Torres et al., 2017; Roda et al., 2020). The cause and progression of Crohn’s disease remains unclear. It has been reported that several factors involve in the cause of Crohn’s disease include genetic susceptibility, environmental factors (e.g., diet), altered gut microbiota, and dysregulated immune system (Torres et al., 2017; Levine et al., 2018; Wark et al., 2020). However, the contribution of genetics together only explained 19%–26% of the hereditary variance of IBD (Peters et al., 2017). Diet is an important factor in the pathogenesis and treatment of IBD (Pascal et al., 2017). Dietary components that are associated with the risk of IBD mainly include dietary fiber, sugar, fat, and protein (Mentella et al., 2020). One large prospective study of the dietary patterns demonstrated the increased protein intake, specifically animal protein in the form of meat or fish correlated with IBD (Jantchou et al., 2010). Some animal studies also showed that high-protein diet had harmful effects on experimental colitis (Lan et al., 2016; Vidal-Lletjos et al., 2019).

One of the mechanisms involved in the pro- or anti-inflammatory effects of diet is the intermediate impact of diet on the composition and metabolic activity of the gut microbiota (Zheng et al., 2020). Microbes can act as intermediate factors in intestinal inflammation (Ramanan et al., 2014). The majority of animal models of colitis require microbiota to develop intestinal inflammation, and there is no evidence of colitis in germ-free mice (Weingarden and Vaughn, 2017; Glassner et al., 2020). A systematic review based on high-quality case-control studies showed that *Escherichia coli* was the most consistent harmful microbiota, whereas the abundance of *Lachnospiraceae* decreased in patients with IBD compared with healthy controls (Pittayanon et al., 2020). It was reported that adherent invasive *Escherichia coli* linked to patients with Crohn’s disease (Palmela et al., 2018; Mirsepasi-Lauridsen et al., 2019). Besides, numerous previous studies indicated that the abundance of *Lachnospiraceae* decreased in patients with IBD (Baumgart et al., 2007; Frank et al., 2007; Lepage et al., 2011; Gevers et al., 2014; Haberman et al., 2014), and *Lachnospiraceae* was a major producer of the short-chain fatty acid (SCFA) in the human gut, which promoted epithelial barrier integrity and inhibited intestinal inflammation (Chen et al., 2017; Sun et al., 2021).

Notably, there is no single microorganism alone can explain the occurrence of IBD, and antibiotics targeting a particular microbe has not shown long-term effectiveness in the treatment of Crohn’s disease (Weingarden and Vaughn, 2017). Indeed, there have been extensive investigations of specific microbes in the intestine with the development of high-throughput sequencing techniques but still limited research concerning the interaction within the microbiota (Coyte et al., 2015). It is crucial to explore the gut microbiota of patients with Crohn’s disease from multiple perspectives and the effect of different diets on the microbiota. To explore the potential impact of altered gut microbiota under HPD on Crohn’s disease, the co-occurring gut microbial network and markers from public databases have been used and HPD mice model was constructed to investigate the changes of composition and function of intestinal microbiome.

## Materials and Methods

### The Data From Public Database

By querying GMrepo^1^ (Wu et al., 2020) for data of gut microbes of healthy individuals and the patients with Crohn’s disease, the gut microbial network was obtained and filtered to show only a portion of the microbial co-occurring network associated with *Escherichia coli*. In addition, the biomarkers related to Crohn’s disease were evaluated by the LEfse algorithm in this website. The MicroPattern database^2^ (Ma et al., 2017a,b) was subsequently used to perform enrichment analysis of microorganisms in different diseases.

### Animals

Female BALB/c mice (3 weeks old, 12–15 g) from Hunan SJA Laboratory Animal Co. Ltd. (Changsha, China) were raised under specific pathogen–free condition and divided into two groups randomly. One group was fed with standard diet (SD), and the other with HPD for 4 weeks (the specific diet information of the two groups of mice is shown in Table 1). After that, the mice were sacrificed through the inhalation of isoflurane. The colon was then incised longitudinally by a sterilized scissor, and the mucus was rubbed by a sterile swab for metagenomic sequencing. The study was approved by the Institutional Ethics Committee for Animal Procedures of the Central South University (No. 2018syclwo0252).

**TABLE 1:** Composition of the experimental diets.

	SD	HPD
Casein (g/kg)	200	593
Cystine (g/kg)	3	3
Starch (g/kg)	397	67
Maltodextrin (g/kg)	132	69
Sucrose (g/kg)	100	100
Fiber (g/kg)	50	50
Fat (g/kg)	70	70
Antioxidants (g/kg)	0.014	0.014
Minerals (g/kg)	35	35
Vitamins (g/kg)	10	10
Choline bitartrate (g/kg)	2.5	2.5

### Metagenomic Sequencing and Taxonomic Classification

DNA concentration was measured by the Qubit^®^ dsDNA Assay Kit in Qubit^®^ 2.0 Flurometer (Life Technologies, CA, United States). DNA (1 μg) per sample was used as input material for the preparation of DNA samples. Sequencing libraries were constructed using NEBNext^®^ Ultra™ DNA Library Prep Kit for Illumina (NEB, United States), and index codes were added to attribute sequences to each sample. Briefly, the DNA sample was sheared by sonication to a size of 350 bp, and then, DNA fragments were end-polished and ligated with the full-length adaptor for Illumina sequencing with further PCR amplification, PCR products were purified (AMPure XP system), and libraries were analyzed for size distribution by Agilent2100 Bioanalyzer and quantified using real-time PCR. The clustering of the index-coded samples was performed on a cBot Cluster Generation System, and next library preparations were sequenced on an Illumina HiSeq platform. Raw sequences were processed to remove low-quality sequences using fastp (Chen et al., 2018) (version 0.21.0) and FastUniq (Xu et al., 2012) (version 1.1.0) to eliminate duplicates in paired short DNA sequence reads in a FASTQ format. The sequences from mice were filtered out using the mice reference genome (mm39) by Bowtie2 (Langdon, 2015) (version 2.3.5). The remaining high-quality reads were used for taxonomic classification by Kraken2 (Wood et al., 2019) (version 2.0.7), utilizing the custom Kraken2 microbiological database including bacteria and fungi with default settings. The read count tables of several levels (e.g., phylum, class, order, family, genus, and species) were generated by Bracken (Lu et al., 2017) to estimate the relative abundance of microorganisms in different samples.

### Metagenomic Assembly, Gene Prediction, and Function Annotation

After quality control, the remaining high-quality reads were assembled by megahit (Li et al., 2015) (version 1.2.9) with default settings. In total, we retrieved 1,418,865 contigs with 254,615,694 base pairs. The contigs greater than 350 bp were used to perform the coding sequence (CDS) prediction with MetaGeneMark (Zhu et al., 2010) (version 3.38). We predicted 801,725 CDSs from the filtered contigs. A gene catalog was established using all predicted genes after de-redundancy by CD-HIT (Fu et al., 2012) and 413,994 genes remained. The read coverage for each gene in different samples was estimated by Salmon (Patro et al., 2017) with mapping raw reads from each sample to sequences in gene catalog. The function annotation for genes in gene catalogs was performed by eggNOG-mapper (Huerta-Cepas et al., 2017) (version 2.0.1). The genes annotated with tax scope in bacteria, fungi, and viruses were remained only to ensure the accuracy of annotations and prevent contamination of host genes. After annotation, a total of 9,684 genes remained. To research the potential functions of these genes, we added up the abundance of genes annotated as the same ortholog (OG) in eggNOG and finally got 7,869 OGs.

### Determination of Gene Host

To determine the host of the predicted genes, we used DIAMOND (Buchfink et al., 2015) (version 2.0.8.146) to align the predicted CDSs to the non-redundant database sub-library from NCBI, which is containing only microorganisms and utilized MEGAN (Arumugam et al., 2019) (version 6.20.11) software to determine its belonging classification of microorganisms. In MEGAN’s algorithm, we used a relatively conservative discrimination method (only genes were determined in the same clades, which were considered to be this clade).

### Detection of Different Species and Association Network Construction

The different species between HPD and SD groups were identified by R package ANCOMBC (Lin and Das Peddada, 2020) (version 1.0.5), and 1,619 different species were detected out from 4,808 species. These different species all passed significance thresholds [need *P* < 0.05, adjustment using false discovery rate (FDR)]. To explore the correlation of different microbes, the co-occurrence network was constructed on the basis of relative abundances of different species. The species with the number of reads < 6 were excluded to avoid unreliable results, and the FastSpar (Watts et al., 2019) (version 1.0) was then used to realize the SparCC network efficiently. Only statistically significant (*P* < 0.05) correlations were considered in network analyses. Afterward, the R package igraph was used to construct the microbial network. To further find the potential identical functional groups between the constructed microbial co-occurrence network and the core bacteria that are critical, we used MCODE (Bader and Hogue, 2003) in Cytoscape (Shannon et al., 2003) with default settings. After that, 101 species were obtained in final co-occurrence networks within 12 clusters, and 10 hub species were identified. The Cytoscape (3.8.0) was used to visualize the network and the sub-networks.

### Discrimination of Difference Orthologs

Mann–Whitney rank test was performed for all OGs, with the calculation of corrected P-value (FDR). The “log2FC” was explained by **l***og***2FC**=**l***og***2**[(**A** + **1**)/(**B** + **1**)], where A and B are the OGs abundance of HPD and SD groups, respectively. The filter condition was FDR < 0.385, *P* < 0.05, and | log2FC| > 2. Finally, 15 OGs were detected. All calculations above were implemented in python (version 3.8.5).

### Short-Chain Fatty Acid Analysis

Short-chain fatty acids (SCFAs) were extracted from mouse feces using acetonitrile: water (1:1) and derivatized using 3-nitrophenylhdyrazones. SCFAs were analyzed on a Jasper HPLC coupled to Sciex 4500 MD system. In brief, individual SCFAs were separated on a Phenomenex Kinetex C18 column (100 × 2.1 mm, 2.6 μm) using 0.1% formic acid in water as mobile phase A and 0.1% formic acid in acetonitrile as mobile phase B. Octanoic acid-1-13C1 purchased from Sigma-Aldrich and Butyric-2, 2-d2 from CDN Isotopes were used as internal standards for quantitation (Li et al., 2019).

### Other Bioinformatics Analysis and Data Visualization

The Multi-Response Permutation Procedure (MRPP) test was implemented in the vegan package (version 2.5-7) under the R platform. The R package mixOmics (version 6.10.9) was used to make partial least-squares discriminant analysis (PLS-DA) model. The data visualization was performed in R (version 3.6.1).

## Results

### *Escherichia coli* and *Lachnospiraceae* Have Potential Negative Correlation in Microbial Gut Co-occurrence Network of Crohn’s Disease

**FIGURE 1:**
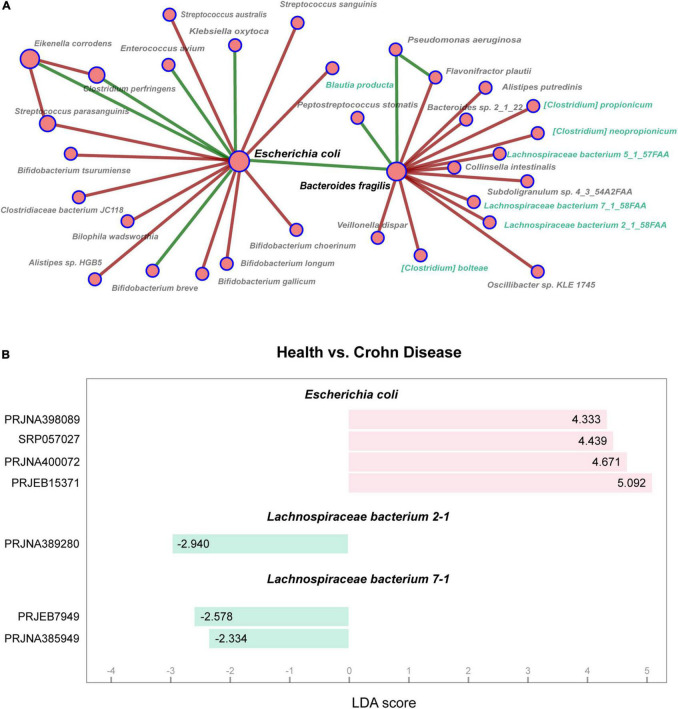
Analysis of gut microbiota in patients with Crohn’s disease. **(A)** Partial of co-occurrence microbial network in patients with Crohn’s disease shows relationships among gut microbes. The node sizes are proportional to the number of connected nodes in the network, and the colors indicate positive (green) or negative (red) correlations. The nodes marked with green belong to *Lachnospiraceae* family. **(B)** Marker taxa are shown between health and Crohn’s disease using LEfSe analysis. Y-axis represents BioProject ID in NCBI, and X-axis represents LDA scores calculated by LEfSe.

To explore the co-occurrence of *Escherichia coli* and other microbes in the intestines of patients with Crohn’s disease, we investigated the gut microbial network in the GMrepo and selected part of gut microbiota network of Crohn’s disease as shown in Figure 1A. It was found that there existed an indirect negative correlation between *Escherichia coli* and *Lachnospiraceae*. In the data sets of multiple studies, *Escherichia coli* was a marker for Crohn’s disease. Whereas *Lachnospiraceae* bacterium 2-1 and *Lachnospiraceae* bacterium 7-1 belonging to *Lachnospiraceae* were markers in the healthy controls (Figure 1B).

### Six Notable Microbes Are Detected Under the Influence of High-Protein Diet

**FIGURE 2:**
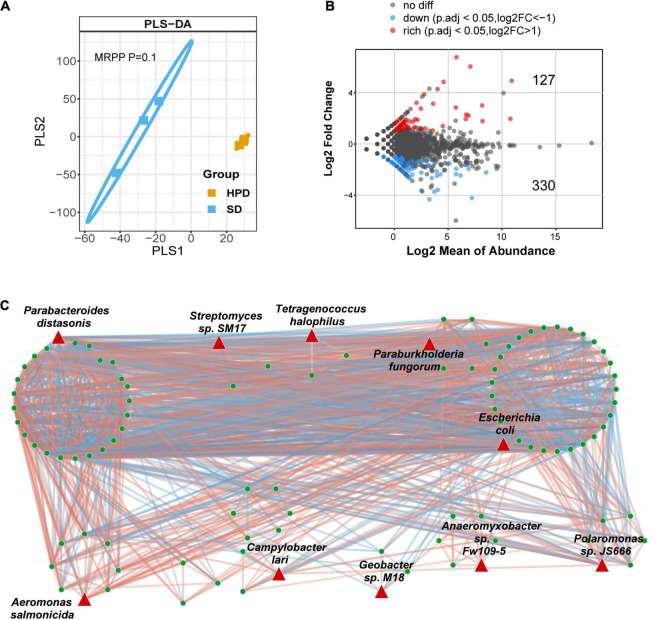
Alteration of intestinal microbial structure in mice fed a high protein diet. **(A)** PLS-DA analysis based on the relative abundance of species reflects the between-subjects (β) diversity across groups, in which the orange circles and blue squares represent HPD and SD groups, respectively (MRPP, *P* = 0.1). **(B)** Volcano plot demonstrates the differential abundance of species between HPD and SD groups. ANCOMBC is used to calculate the P-values. Points are colored according to the number of log2FC if they passed significance thresholds. (Adjusted *P* < 0.05, adjustment using FDR). **(C)** The microbial co-occurrence network where different nodes indicate different species is constructed. Red links indicate positive covariation between two individual nodes, whereas blue links indicate negative covariation. The hub species are marked by red triangles.

**FIGURE 3:**
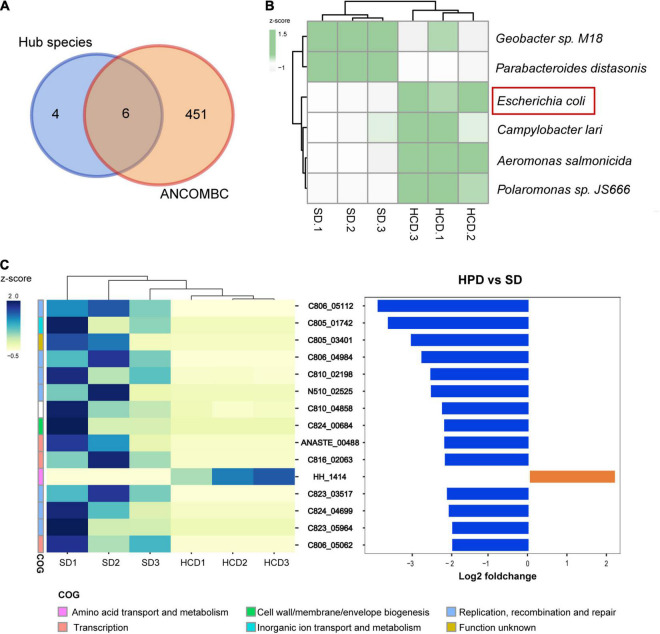
Gut microbiota and OGs with significantly different abundance under high protein diet. **(A)** Venn diagram shows the number of shared and unique species for the hub species and ANCOMBC. **(B)** The heatmap graph shows the relative abundance of six taxa detected in Figure A. The colors from white to green represent the degree of difference. **(C)** There are 15 OGs detected with the threshold value [Mann–Whitney rank test, *P* < 0.05, | log2FC| > 2]. They demonstrate functional genetic alterations in intestinal microbes with high-protein diet intervention.

To explore the impact of HPD on the intestinal flora, metagenomic sequencing was performed in mice from HPD and SD group, followed by mapping of various levels of intestinal microbial composition. The complex composition of gut microbes is presented on a two-dimensional plane using a supervised learning PLS-DA model. The composition of the gut microbes in HPD group converged, whereas it was more dispersed in the other group (Figure 2A). Similarly, according to MRPP test, there was significant difference of between-subjects (β) diversity between HPD and SD groups (*P* = 0.1). To accurately identify the impact of HPD on the gut microbes, ANCOMBC algorithm was applied in strict screening conditions (refer to Materials and Methods) to screen out 457 different microbes (Figure 2B). Next, a gut microbial co-occurrence network was constructed on the basis of the results of ANCOMBC. There were 12 clusters and 10 seed nodes, which were considered as hub species in the network (Figure 2C). Considering the statistical differences between groups and the topology of the network, the intersection of the two results was taken and presented in Figure 3A. Finally, six notable microbes were detected out and their relative abundance were shown in Figure 3B.

### The Functional Changes of Microbiome Under the Influence of High-Protein Diet

The top 15 different OGs between groups sorted by log2FC were shown in Figure 3C. Among them, only the HH_1414 was enriched in HPD group, which was annotated as “Amino acid transport and metabolism” in COG database and was considered as a subunit of tryptophan synthase in eggNOG database. However, there were eight OGs related to life activities such as replication annotated as “Replication, recombination and repair” in COG database. The remaining three other types of OGs were annotated in COG as “Transcription,” “Cell wall/membrane/envelope biogenesis,” and “Inorganic ion transport and metabolism,” respectively. The specific descriptions for each OG were annotated in Table 2.

**TABLE 2:** Different eggNOG orthologs annotation.

eggNOG orthologs	eggNOG description	EC
C806_05112	Transposase	
C805_01742	ABC transporter transmembrane region	
C805_03401	Tetratricopeptide repeat	
C806_04984	DDE superfamily endonuclease	
C810_02198	Uncharacterized protein family (UPF0236)	
C810_04858	DDE superfamily endonuclease	
N510_02525	Psort location cytoplasmic	
C824_00684	DDE superfamily endonuclease	
ANASTE_00488	WYL domain	
C816_02063	Psort location cytoplasmic	
HH_1414	The alpha subunit is responsible for the aldol cleavage of indoleglycerol phosphate to indole and glyceraldehyde 3- phosphate	Tryptophan synthase
C823_03517	Transposase IS200 like	
C824_04699	Psort location cytoplasmic	
C823_05964	Psort location cytoplasmic	
C806_05062	Bacterial regulatory proteins, tetR family	

### The Antagonistic Relationship Between *Helicobacter* and *Lachnospiraceae* Under the Influence of High-Protein Diet

**FIGURE 4:**
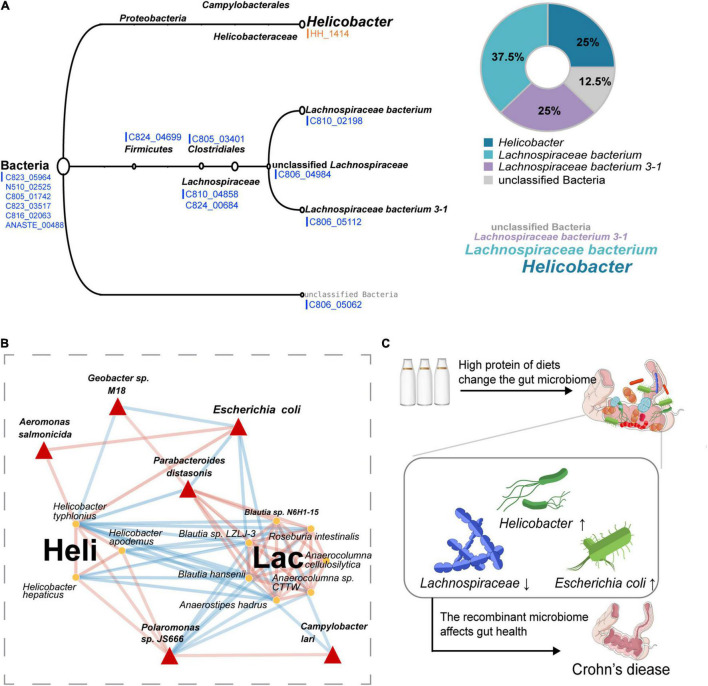
Sources of differential OGs and the symbiotic relationship of these microbes under HPD. **(A)** The classification levels of 15 different OGs are shown, orange represents those enriched in HPD group, and blue represents the enrichment in SD group. **(B)** A sub-network containing *Helicobacter* genus, *Lachnospiraceae* family, and the filtered six hub species is extracted from the previous microbial co-occurrence network. Red links indicate positive covariation between two individual nodes, whereas blue links indicate negative covariation. The hub species are marked by red triangles. In addition, “Heli” represents *Helicobacter*, and “Lac” represents *Lachnospiraceae*. **(C)** A hypothetical pattern that HPD affects Crohn’s disease by altering the composition of gut microbes is proposed.

A rigorous LCA algorithm in MEGAN was further applied to determine the source of these functional genes. It was noting that the HH_1414 OG related to tryptophan synthesis was from *Helicobacter*, whereas most of the other OGs with reduced abundance came from *Lachnospiraceae* (Figure 4A). The relative abundance of *Helicobacter* in HPD group was significantly higher than that in SD group, whereas it was opposite for *Lachnospiraceae* (Supplementary Figure 1). Next, the species belonging to *Helicobacter* and *Lachnospiraceae* including their related hub species in microbial co-occurrence network were extracted to construct a sub-network, which was displayed in Figure 4B. The results suggested that the species from the same clusters tended to co-occur, whereas the relationship between two clusters showed a negative correlation.

### Microbes in the Sub-Network Are Consistent With Crohn’s Disease

To check whether the microorganisms in the microbial sub-network under the influence of HPD (Figure 4B) affected Crohn’s disease, the MicroPattern database was used and these microbes were enriched in Crohn’s disease (Supplementary Figure 2). In addition, three microorganisms (*Anaerostipes hadrus*, *Roseburia intestinalis*, and *Parabacteroides distasonis*) in the sub-network were detected as healthy markers (Supplementary Figure 3). On the basis of the above research, we proposed a putative pattern that excessive protein intake would lead to changes in the structure of gut microbes, which, in turn, affect the occurrence and development of Crohn’s disease, as shown in Figure 4C.

## Discussion

Dietary intervention with specific characteristics can be important during the treatment of the inflammatory process in patients with IBDs (Barros et al., 2021). For patients with IBD, high fermentable oligosaccharides, disaccharides, momosaccharides, and polyols (FODMAP) diet contributes to higher gastrointestinal dysfunction symptoms such as abdominal pain, bloating, and urgency (Cox et al., 2017), whereas low-FODMAP diet is effective in relieving gut symptoms and reducing the fecal abundance of microbes with immune regulation function (Cox et al., 2020). A study of 60 patients with IBD showed improved symptom scores and fecal calprotective protein in the low-FODMAP diet subgroup compared to the SD (Bodini et al., 2019). Mediterranean diets, diets high in fruits, vegetables, and other plant foods, as well as high-fiber diets (including fiber supplementation) are associated with reduced levels of inflammation (King et al., 2007; Smidowicz and Regula, 2015; Wagenaar et al., 2021).

Our study revealed that changes in the gut under the influence of HPD, which were consistent with Crohn’s disease. It suggested that HPD may have potential impact on Crohn’s disease by affecting structural changes in gut microbes. In our experiment, as for the composition of gut microbiota, *Escherichia coli* was detected as notable specie and significantly elevated in HPD group; meanwhile, its abundance was also elevated in the patients with Crohn’s disease. This is consistent with previous reports. *Escherichia coli* was enriched in intestines of patients with Crohn’s disease (Gevers et al., 2014; Palmela et al., 2018) and had a high potential to induce Crohn’s disease (Mirsepasi-Lauridsen et al., 2019; Nagayama et al., 2020).

To investigate the functional change of gut microbiome, firstly, we found that HH_1414 annotated as tryptophan synthase was enriched in HPD group and mainly contributed by *Helicobacter* while reduced OGs mainly caused by *Lachnospiraceae*. Tryptophan was found to be related to Crohn’s disease (Jansson et al., 2009; Jacobs et al., 2016). Some species of *Helicobacter* were considered to be detrimental in intestine. For instance, it was reported that IBD was driven by reactive T cells caused by *Helicobacter hepaticus* (Xu et al., 2018) and *Helicobacter typhlonius* was a key disease trigger to promote and aggravate IBD (Chichlowski et al., 2008; Powell et al., 2012). For *Lachnospiraceae*, it can influence the host epithelium and mucosal immune system by enriching in proximity to the mucosa (Nava et al., 2011; Van den Abbeele et al., 2013; Riva et al., 2019). The genomic analysis of *Lachnospiraceae* suggested a significant role of using diet-derived starch and other sugars to promote the production of SCFAs (Byndloss et al., 2017; Vacca et al., 2020). SCFAs generated by intestinal microbial metabolism may reduce the risk of Crohn’s disease through increasing mucosal immune tolerance (Schirmer et al., 2019). However, no significant differences in the content of SCFAs in feces were found in our study (Supplementary Figure 4). This may be due to the cross-feeding of gut microbes (D’Souza et al., 2018) or due to the limitation of sample size. Our previous study found that the antibiotics can reduce damage of mucus layer caused by a high-protein diet (Chen et al., 2021). However, gut microbes are a complex community in which the altruistic behavior and community effects of drug-resistant bacteria need to be taken into account (Lee et al., 2010; Frost et al., 2018). It was reported that *Lachnospiraceae* can contribute to the microbiota-mediated colonization resistance against drug-resistant pathogens through conversion of primary to secondary bile acids (Buffie et al., 2015; Studer et al., 2016). Besides, the tissue samples from the patients with Crohn’s disease were characterized by decrease in specific genera from families *Ruminococcaceae* and *Lachnospiraceae* (Tyler et al., 2016). Secondly, our study revealed that an alteration of the microbiota in the sub-network was constructed and that *Anaerostipes hadrus*, *Roseburia intestinalis*, and *Parabacteroides distasonis* were detected as markers of healthy controls. *Parabacteroides distasonis* was a node between *Helicobacter* and *Lachnospiraceae* in the sub-network, which had a negative correlation with the former and positively correlated with the latter in our study. Moreover, it was reported that *Parabacteroides distasonis* could contribute to repair the integrity of the intestinal wall and was closely related to the metabolism of bile acids (Wang et al., 2019). Bile acid metabolites were generated by bacteria from host-produced bile acids, which, however, were reduced in patients with IBD (Schirmer et al., 2019). *Roseburia intestinalis* belonging to *Lachnospiraceae* was reported to reduce disease activity index scores and alleviate intestinal mucosal epithelial injury in a mouse model of colitis (Shen et al., 2018). *Anaerostipes hadrus* was also a bacterium from *Lachnospiraceae*, which played an important role in inositol catabolism-butyrate biosynthesis pathway (Zeevi et al., 2019). Butyrate was also a type of SCFAs, which can promote the development of regulatory T cells and continuously strengthen the mucosal barrier. Consequently, the structure of gut microbe’s changes under excessive protein maybe closely related to Crohn’s disease.

Nevertheless, there are some limitations in our study, for instance, larger clinical cohort and lack of differences in gut microbes in rodents and exploration of humans, which need to be covered in the future. In inclusion, only female mice were considered in our experiments, which is unclear for the bias of the experimental results, and some studies have reported that sex has not been clarified for the occurrence of IBD (Severs et al., 2018; Greuter et al., 2020). Our present study has taken the first step toward bringing a new perspective to elucidate the complex network between Crohn’s disease and HPD using the gut microbiota as a bridge. However, more metabolites and immune-related cytokines in addition to SCFAs should be considered in the next step, which could help to establish a link between gut microbes and host immune indicators under the influence of HPD. In addition, the functional studies on mice are also needed to confirm our proposed effect of altered gut microbiota on Crohn’s disease.

## Conclusion

In this work, we focused on the potential impact of altered gut microbiota on Crohn’s disease under HPD, and constructed a mouse model of HPD to study changes in the composition and function of the gut microbiota. Our results revealed a consistency in the alteration of gut microbes in HPD and Crohn’s disease. In the meantime, we proposed a pattern of the effect of altered gut microbes under HPD on Crohn’s disease on the basis of the co-occurrence relationship of gut microbes. In addition, we explained the reason for altered functions of gut microbes under HPD. Therefore, our study provided new ideas for explaining how HPD affect Crohn’s disease. For future studies, combining multi-omics approaches together may better explain the effect of HPD on the alteration of gut microbes on the development of IBD. In addition, the use of gut microbial interventions to verify the therapeutic effect on IBD should be further validated.

## Data Availability Statement

The raw sequence data reported in this paper have been deposited in the Genome Sequence Archive (Genomics, Proteomics and Bioinformatics 2017) in National Genomics Data Center (Nucleic Acids Res 2021), China National Center for Bioinformation/Beijing Institute of Genomics, Chinese Academy of Sciences, under accession number CRA005503, that are publicly accessible at https://bigd.big.ac.cn/gsa.

## Ethics Statement

The animal study was reviewed and approved by Institutional Ethics Committee for animal procedures of the Central South University (No.2018syclwo0252).

## Author Contributions

ZY and SC conceived the study. YZ analyzed the data. LLC performed the experiments. YZ, JH, and LLC wrote the manuscript. All authors contributed to the article and approved the submitted version.

## Conflict of Interest

The authors declare that the research was conducted in the absence of any commercial or financial relationships that could be construed as a potential conflict of interest.

## Publisher’s Note

All claims expressed in this article are solely those of the authors and do not necessarily represent those of their affiliated organizations, or those of the publisher, the editors and the reviewers. Any product that may be evaluated in this article, or claim that may be made by its manufacturer, is not guaranteed or endorsed by the publisher.

## Footnotes

1
https://gmrepo.humangut.info

2
http://www.cuilab.cn/micropattern
