# Supplementary material for: Exploration of the Potential Relationship Between Gut Microbiota Remodeling Under the Influence of High-Protein Diet and Crohn’s Disease
Source: Front Microbiol. 2022 Mar 3;13:831176. doi: 10.3389/fmicb.2022.831176 (PMC8927681; doi:10.3389/fmicb.2022.831176)
Supplement: Supplementary file 1 [file Data_Sheet_1.docx]

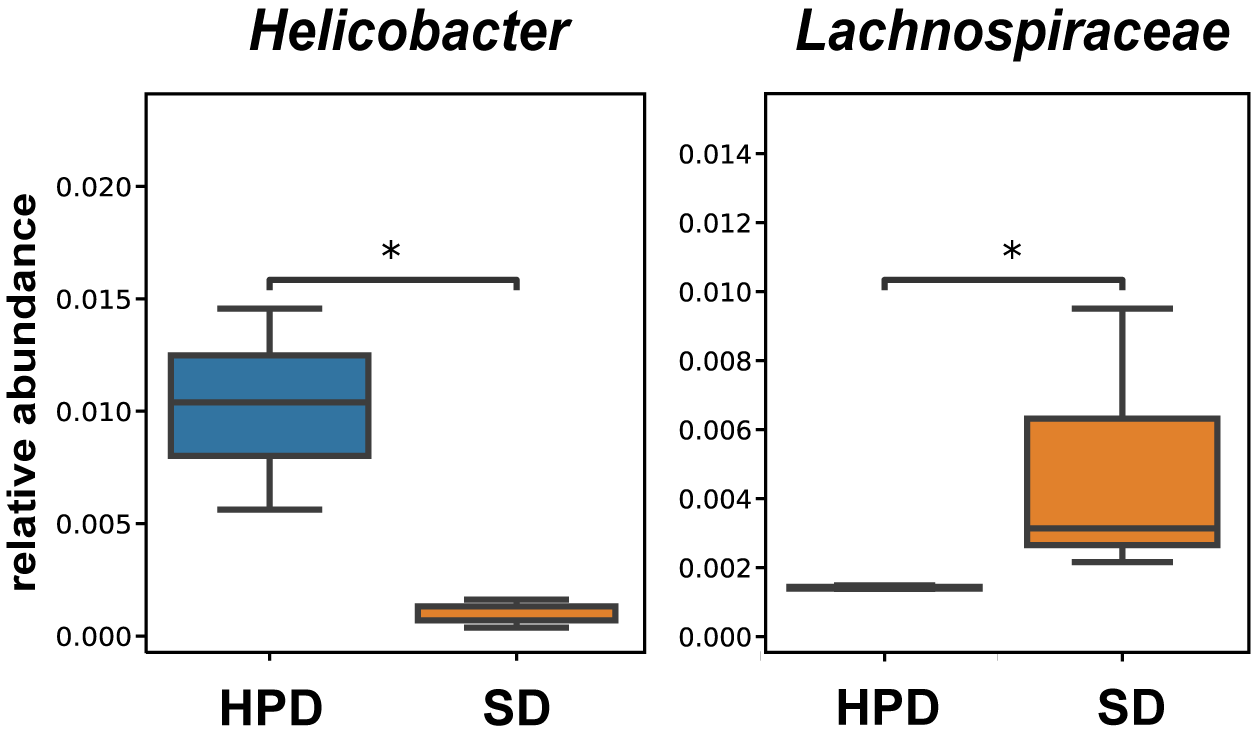


**Supplementary Figure 1.** The box plots showed the relative abundance of differences in each group. (Kruskal test, the asterisk means P < 0.05).


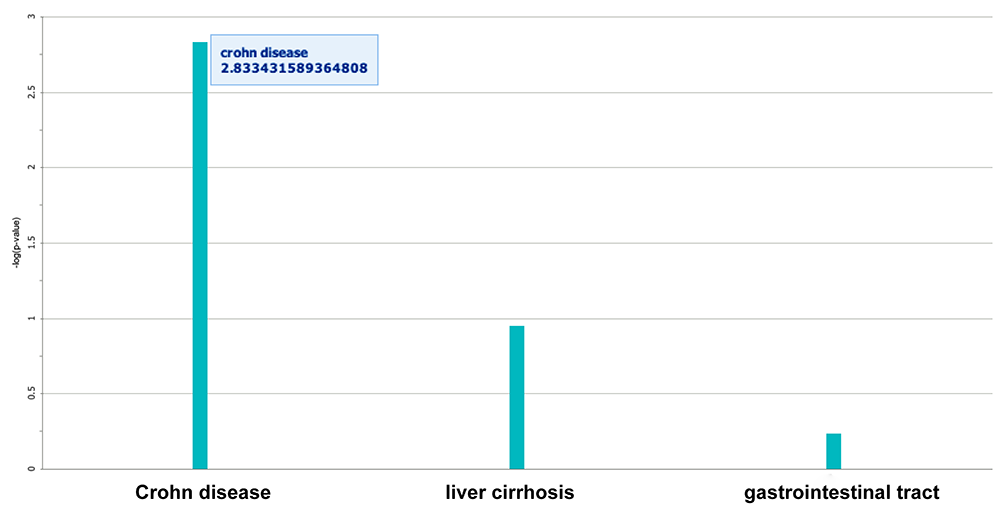


**Supplementary Figure 2.** The barplot from Micropattern (http://www.cuilab.cn/micropattern) by using hypergeometric distribution test show the target microbiome are enriched in Crohn’s disease.


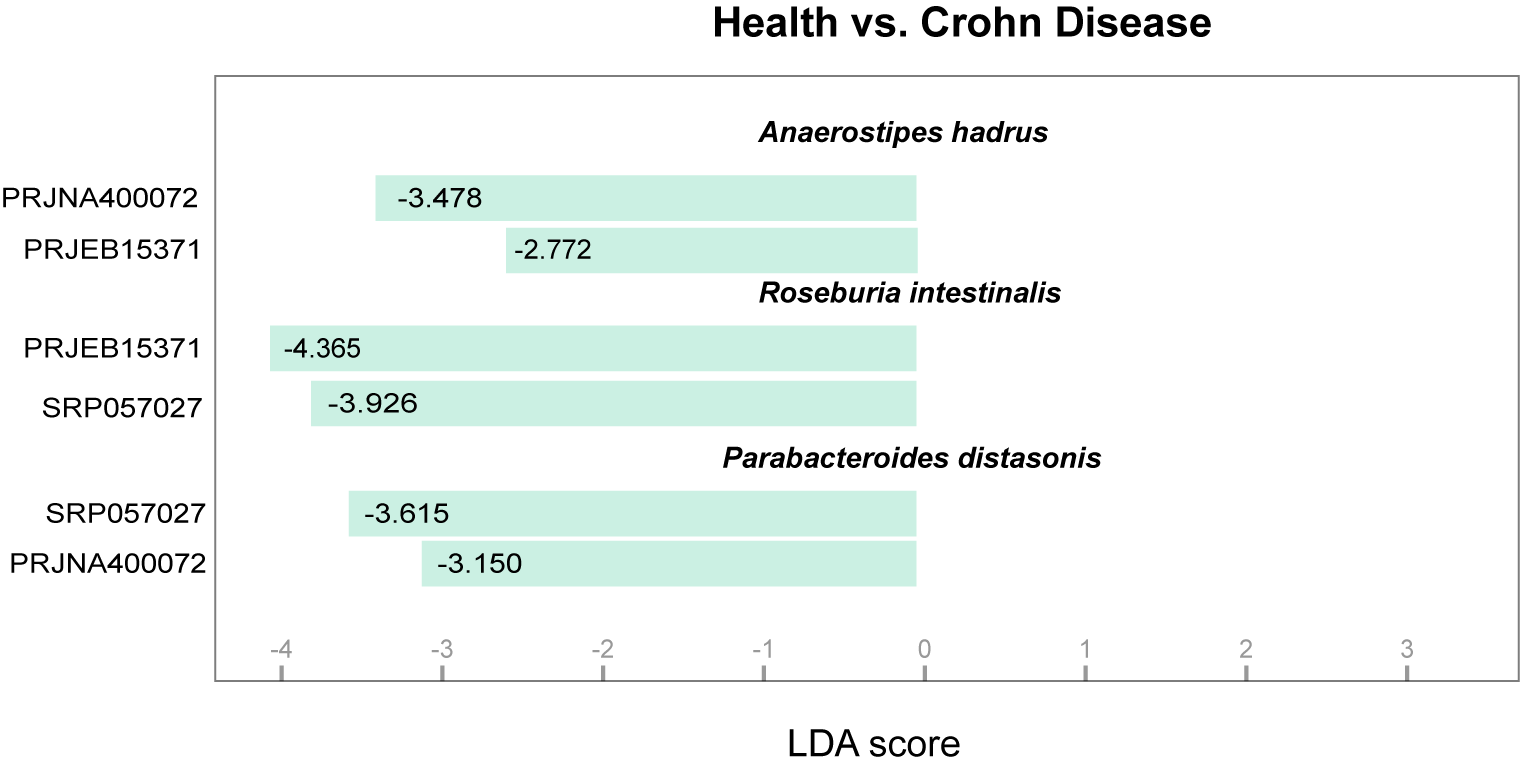


**Supplementary Figure 3.** The barplot of marker taxa showed sigificant differences in relative abundances between health and crohn’s disease, and they were identified using LEfSe (Linear discriminant analysis Effect Size) analysis. Y axis represents BioProject ID in NCBI,and X axis represents LDA scores calculated by LEfSe (The results are from GMRepo, <https://gmrepo.humangut.info>).


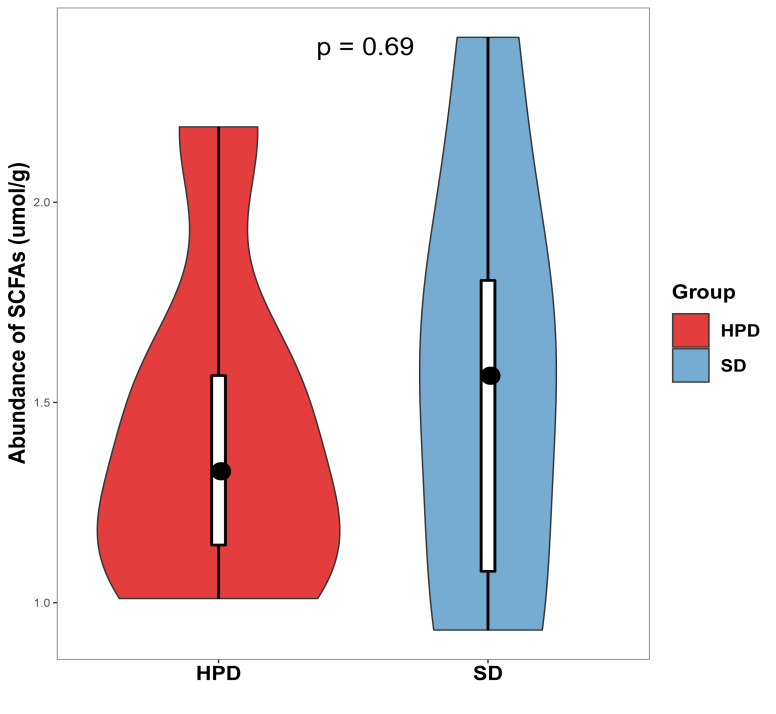


**Supplementary Figure 4.** The box plot showed the abundance of SCFAs in each group. The SCFAS includes acetic acid, propionic acid, isobutyric acid, butyric acid, 2-methyl-butyric acid, valeric acid, isovaleric acid and hexanoic acid (Wilcoxon rank sum test).
